# “LRRK2: Autophagy and Lysosomal Activity”

**DOI:** 10.3389/fnins.2020.00498

**Published:** 2020-05-25

**Authors:** Marta Madureira, Natalie Connor-Robson, Richard Wade-Martins

**Affiliations:** ^1^Department of Physiology, Anatomy and Genetics, Oxford Parkinson’s Disease Centre, University of Oxford, Oxford, United Kingdom; ^2^Graduate Program in Areas of Basic and Applied Biology (GABBA), Instituto de Ciências Biomédicas de Abel Salazar, Universidade do Porto, Porto, Portugal

**Keywords:** Parkinson’s disease, LRRK2, autophagy, lysosomes, kinase, GTPase, G2019S, R1441C

## Abstract

It has been 15 years since the *Leucine-rich repeat kinase 2* (*LRRK2*) gene was identified as the most common genetic cause for Parkinson’s disease (PD). The two most common mutations are the LRRK2-G2019S, located in the kinase domain, and the LRRK2-R1441C, located in the ROC-COR domain. While the LRRK2-G2019S mutation is associated with increased kinase activity, the LRRK2-R1441C exhibits a decreased GTPase activity and altered kinase activity. Multiple lines of evidence have linked the LRRK2 protein with a role in the autophagy pathway and with lysosomal activity in neurons. Neurons rely heavily on autophagy to recycle proteins and process cellular waste due to their post-mitotic state. Additionally, lysosomal activity decreases with age which can potentiate the accumulation of α-synuclein, the pathological hallmark of PD, and subsequently lead to the build-up of Lewy bodies (LBs) observed in this disorder. This review provides an up to date summary of the LRRK2 field to understand its physiological role in the autophagy pathway in neurons and related cells. Careful assessment of how LRRK2 participates in the regulation of phagophore and autophagosome formation, autophagosome and lysosome fusion, lysosomal maturation, maintenance of lysosomal pH and calcium levels, and lysosomal protein degradation are addressed. The autophagy pathway is a complex cellular process and due to the variety of LRRK2 models studied in the field, associated phenotypes have been reported to be seemingly conflicting. This review provides an in-depth discussion of different models to assess the normal and disease-associated role of the LRRK2 protein on autophagic function. Given the importance of the autophagy pathway in Parkinson’s pathogenesis it is particularly relevant to focus on the role of LRRK2 to discover novel therapeutic approaches that restore lysosomal protein degradation homeostasis.

## Introduction

Parkinson’s disease (PD) is the second most common neurodegenerative disorder worldwide, with more than 4 million people over 50 affected by the disease, with a projection for the number of individuals with PD to double by 2030 (Dorsey et al., 2007). PD is characterised by a loss of DAn in the SNpc region of the midbrain as well as their projections to the striatum. This specific dopaminergic neurodegeneration within an important regulator of voluntary movements results in the hallmark clinical symptoms of PD such as bradykinesia, resting tremor, and muscle rigidity (Antony et al., 2013). Moreover, *post-mortem* analysis reveals the presence of α-synuclein protein aggregates, known as LBs, throughout the brain (Spillantini et al., 1997). Alpha-synuclein forms the main component of LBs and is thought to spread to interconnected brain regions in a prion-like manner, a process that is currently not fully understood (Wang and Hay, 2015).

Currently, 5–10% of patients present with a familial form of PD, illustrating how research into these genes and their neuropathological pathways is vital. Familial mutations have been described in a number of genes including *SNCA* and *LRRK2* which are responsible for autosomal dominant PD forms, while mutations in *Parkin*, *PINK1*, *DJ-1*, and *ATP13A2* are accountable for autosomal recessive PD (Klein and Westenberger, 2012). GWAS have uncovered numerous low-risk susceptibility variants for sporadic PD, namely in the *LRRK2*, *GBA*, *MAPT*, *Parkin*, *PINK1*, *DJ-1*, and *VPS35* loci (Hardy, 2010; Singleton et al., 2013; Nalls et al., 2014). Moreover, the study identified a *LRRK2* variant in a non-coding region (rs76904798h), which confers around 15% increased risk of developing sporadic PD (Nalls et al., 2014). A recent meta-analysis of GWAS has reported a list of 17 additional loci associated with disease, altogether implicating pathways such as neuronal survival, neuroinflammation, vesicle trafficking, mitochondria metabolism, autophagy, and lysosomal function with PD (Chang et al., 2017).

Two recent studies independently showed that *GBA* mutation carriers can also carry the *LRRK2-G2019S* mutation, with no impact on age of onset (Yahalom et al., 2019; Blauwendraat et al., 2020). However, when compared to non-carriers, *LRRK2-G2019S* carriers displayed higher activity levels of GCase, the lysosomal membrane enzyme encoded in the *GBA* gene that cleaves the glycolipid glucosylceramide. Since *GBA* mutations are linked to reduced GCase activity and more aggressive PD pathology, it was hypothesised *LRRK2* mutations could have a protective effect on *GBA* mutation carriers through a mechanism that still remains unknown (Alcalay et al., 2015). Contrary to this observation, and importantly in iPSC-derived DAn, *LRRK2-G2019S* and *LRRK2-R1441C* patients showed lower GCase activity, which was then increased after treatment with the LRRK2 inhibitor MLi-2, in neurons with either *LRRK2* or *GBA* mutations (Ysselstein et al., 2019). It is hypothesised that reduced GCase activity leads to impaired lysosomal function and therefore accumulation of insoluble α-synuclein (Beavan and Schapira, 2013). Moreover, a recent report showed that LRRK2 inhibitor MLi-2 rescued lysosomal pH levels and corrected cathepsin B activity in *GBA* mutant knock-in astrocytes (Sanyal et al., 2020). Taken together, these results suggest an interplay between GBA and LRRK2, albeit still not fully understood, that paves the way for more research to be conducted on this subject.

The first familial mutation to be linked to PD was the A53T mutation in the α*-synuclein* (*SNCA*) gene which was identified in an Italian family (Polymeropoulos et al., 1997; Hardy, 2010). The A53T mutation in α-synuclein has been associated with altered autophagy and mitochondrial dysfunction (Smith et al., 2005; Pupyshev et al., 2018). Since then, multiple rare genetic alterations such as duplication, triplication and other point-mutations such as A30P, E46K, H50Q, and G51D, have been reported in the *SNCA* gene. *SNCA*-related PD is usually associated with early onset disease with a more rapid progression and with dose dependent effects on both of these outcomes (Wang and Hay, 2015; Schneider and Alcalay, 2017; Zeng et al., 2018). Although the exact role of α-synuclein remains elusive, numerous pathological mechanisms, such as synaptic dysfunction, ER-Golgi trafficking disruption, ER stress, Golgi fragmentation and perturbation of the ALP have been associated with α-synuclein mutations (Colla et al., 2012; Chung et al., 2013; Ryan et al., 2013; Wang and Hay, 2015; Zambon et al., 2019). Other PD associated genes have also been implicated in the autophagy and mitophagy pathways including *GBA*, *VPS35*, *ATP13A2*, *PINK1*, and *Parkin* (Ramirez et al., 2006; Vives-Bauza et al., 2010; Zavodszky et al., 2014; Fernandes et al., 2016; Taguchi et al., 2017).

This review focuses on the role of LRRK2 in autophagy. Different LRRK2 models have been widely used and show a cell-specific role for this protein, as well as phenotypic alterations related to the autophagic flux when LRRK2 is mutated. Impaired autophagy leads to alterations in lysosomal degradation that could be linked to accumulation of misfolded proteins that form aggregates and lead to neurodegeneration.

## LRRK2 and Parkinson’s Disease

Mutations in the Leucine-Rich Repeat Kinase 2 (*LRRK2*) gene, located in the *PARK8* loci, are the most common mutations found in familial autosomal dominant PD (Paisán-Ruíz et al., 2004; Zimprich et al., 2004; Singleton et al., 2013). Due to their similar age of onset, symptom progression and neuropathology *LRRK2*-PD patients cannot be clinicopathologically distinguished from idiopathic patients. LRRK2 is mainly considered to be a cytoplasmic protein, but it can also be found on organelle membranes, such as the mitochondria and lysosomes (Orenstein et al., 2013). Mutations in the *LRRK2* gene account for 2 to 40% of PD cases, depending on populations (Klein and Westenberger, 2012). The two most common mutations, G2019S and R1441C, account for up to 10 and 2.5% of sporadic PD cases, respectively, depending on population group. The difference in frequency between the two mutations may be explained by incomplete, age-dependent penetrance. The G2019S mutation presents a penetrance ranging from 17% at 50 years old to 85% at 70 years old, and the R1441C mutation presents with more severe phenotypes (Healy et al., 2008; Kluss et al., 2019). These findings along with the evidence from GWAS, showing LRRK2 variants impact on the risk of developing PD, show how understanding the role of LRRK2 in PD pathology will be critical to fully comprehend both familial and sporadic forms of disease.

LRRK2 comprises a large homodimeric protein (285 kDa) that is ubiquitously expressed, with the highest levels of LRRK2 being detected in the kidneys, lungs, and brain. Although the role of LRRK2 is not yet fully defined, it has several functional domains including an ARM region, ANK region, and a leucine-rich repeat (LRR) domain, which are important for mediating protein-protein interactions. There is also a ROC-COR domain which consists of a GTPase of the ROCO family. The C-terminal of LRRK2 contains a functional kinase MAPKKK-like domain, regulated by the GTPase activity of LRRK2, and also a WD-40 domain that regulates protein-protein interactions (Guaitoli et al., 2016). Therefore, LRRK2 has both kinase and GTPase function and can also operate as a scaffolding unit in signalling pathways (Paisán-Ruíz et al., 2004; Outeiro et al., 2019). LRRK2 has been reported to phosphorylate several substrates, including GTPases of the Rab superfamily Rab3, Rab5, Rab7L1, Rab8, Rab10, Rab12, Rab29, and Rab32, indicating a role in endosomal and vesicle trafficking pathways (Dodson et al., 2012; MacLeod et al., 2013; Beilina et al., 2014; Cho et al., 2014; Yun et al., 2015; Steger et al., 2016; Connor-Robson et al., 2019). LRRK2 also interacts with microtubules, suggesting a role in cytoskeleton dynamics and neurite outgrowth (Godena et al., 2014; Parisiadou et al., 2014). Studies in LRRK2 mutant models also implicate a role in mitochondria morphology and homeostasis (Yue et al., 2015). LRRK2 regulates pathways in immune cells, such as cytokine release and phagocytosis [reviewed by Wallings and Tansey (2019)]. More recently, LRRK2 has been described to regulate nuclear envelope integrity by interacting with lamin A/C (Shani et al., 2019).

The G2019S mutation, located in the kinase domain, increases the kinase activity of LRRK2 whereas the R1441C mutation, located in the GTPase domain, decreases GTPase activity (Chen and Wu, 2018; West et al., 2005; Lewis et al., 2007). There is evidence suggesting LRRK2-R1441C increases kinase activity (West et al., 2005), yet other reports suggest it does not directly enhance kinase activity (Lewis et al., 2007; Nichols et al., 2010; Steger et al., 2016), thus indicating this effect is still unclear. As discussed above, LRRK2 likely has numerous functions and disruption to its normal physiological roles would result in a broad array of phenotypes within cellular structures. For instance, pathogenic LRRK2 leads to impairment of late stages of endocytosis, trafficking to lysosomes and synaptic vesicle endocytosis (Gómez-Suaga et al., 2012; Rivero-Ríos et al., 2016; Connor-Robson et al., 2019). LRRK2-G2019S models present increased sensitivity to mitochondrial toxins and accumulation of damaged mitochondria while both LRRK2-G2019S and LRRK2-R1441C cause increased mitochondrial fragmentation, suggesting a toxic gain-of-function phenotypic alteration (Mortiboys et al., 2010; Ramonet et al., 2011; Wang et al., 2012; Karuppagounder et al., 2016). Both LRRK2-G2019S and LRRK2-R1441C seem to alter actin cytoskeleton stability and LRRK2-R1441C shows disruption of microtubule-dependent organelle and vesicle transport (Godena et al., 2014; Parisiadou et al., 2014; Caesar et al., 2015). LRRK2-G2019S has reduced interaction with lamin A/C, causing nuclear lamina disorganisation and leakage of nuclear proteins in a loss-of-function manner (Shani et al., 2019). Considering the many potential roles of LRRK2 it is important to understand how it plays a role in each pathway. Studies focused in specific cellular contexts will help uncover the full extent of mutant LRRK2 effects.

## Links Between Parkinson’s Disease and Autophagy

Focused investigation into LRRK2 mutations revealed the first evidence for impaired autophagy and lysosomal dysfunction in cells (Plowey et al., 2008; Alegre-Abarrategui et al., 2009; Gómez-Suaga et al., 2012). Autophagy can be defined as the process that regulates recycling of cellular components by degrading dysfunctional or damaged proteins and organelles. There are several types of autophagy, macroautophagy, microautophagy, CMA, and the recently discovered precision autophagy (Cuervo et al., 2004; Kimura et al., 2015; Manzoni and Lewis, 2017). Contrary to macroautophagy, CMA requires chaperone protein Hsc70 to recognise the target substrate through KFERQ-like motifs. Subsequently, Hsc70 binds to lysosomal protein LAMP2A to internalise substrates, which are then degraded by cathepsins. CMA is largely responsible for α-synuclein clearance, since α-synuclein contains a recognition motif, and is translocated into lysosomes for degradation (Cuervo et al., 2004). The physiological role of LRRK2 and dysfunction in CMA have been recently reviewed by Berwick et al. (2019). It is reported that LRRK2-G2019S acts on LAMP2A and blocks CMA, affecting lysosomal degradation of proteins and precipitating the accumulation of α-synuclein in neuronal cells (Orenstein et al., 2013). In concordance, both *LRRK2-G2019S* iPSC-derived astrocyte cultures and *LRRK2-R1441C* knock-in mouse embryonic fibroblasts showed decreased CMA levels (di Domenico et al., 2019; Ho et al., 2019). In parallel to macroautophagy, these effects will undoubtedly contribute to PD pathology through lysosomal damage.

**FIGURE 1:**
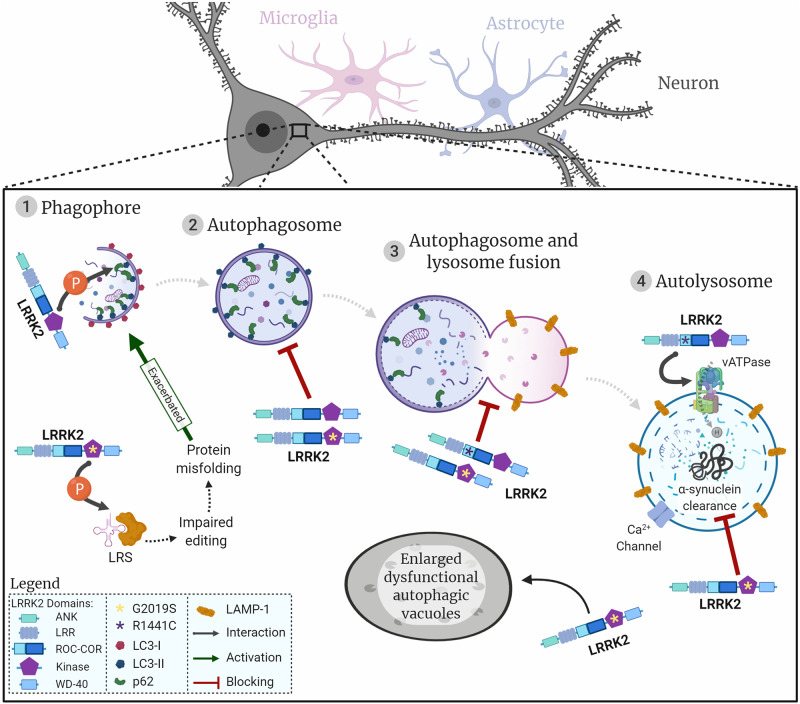
LRRK2 and the neuronal autophagy-lysosomal pathway. Representation of the autophagic process in neuronal cells, initiated with the generation of the phagophore surrounding the cargo to be degraded (1). Once the cargo is fully encapsulated by a bi-layered membrane the autophagosome (2) fuses with the lysosome (3) to produce the autolysosome (4). Proteins/organelles are degraded via lysosomal enzymes. The diagram shows how LRRK2 wildtype and the two most common LRRK2 mutations are likely to impact autophagic flow and lysosomal protein degradation. LRRK2 is represented by its respective domains (ANK, LRR, ROC, COR, Kinase, and WD-40). LRRK2 mutations are represented by asterisks in the respective domains where they are located. G2019S, yellow asterisk in kinase domain; R1441C, purple asterisk in ROC domain; interactions represented by arrows; flow of autophagic phases represented by dashed arrows.

In this review we focus on macroautophagy (henceforth referred to as autophagy). Briefly, cells will respond to certain conditions, such as starvation, through signalling pathways to initiate autophagy. This triggers the formation of the phagophore around the cargo to be degraded, and when the encapsulation is complete it forms an autophagosome. The autophagosome will then fuse with a lysosome (autolysosome) where the cargo will be degraded by lysosomal enzymes (as summarised in Figure 1).

The main regulators of autophagy are mammalian/mechanistic target of rapamycin complex 1 (mTORC1), AMPK, and phosphoinositol 3 kinase (PI3K)/Beclin-1 that act to activate or repress the formation of autophagic vesicles in response to cellular conditions (Noda and Ohsumi, 1998; Meley et al., 2006; Chan et al., 2007; Alers et al., 2012). To initiate autophagy, mTORC1 is inhibited, thus reducing mTORC1-dependent phosphorylation of ULK1 which consecutively switches to an active state. Subsequently, ULK1 activation stimulates phagophore formation (Kamada et al., 2000). Beclin-1 and the vacuolar sorting protein 34 (VPS34), together with other proteins, form a complex that is recruited to the phagophore to stabilise ULK. VPS34 converts phosphoinositol into phosphoinositol-3-phosphate [PI(3)P] which in turn binds to WIPI proteins, to recruit autophagy-related proteins (Atg) (Behrends et al., 2010; Dikic and Elazar, 2018). The ubiquitin signal in tagged proteins binds to p62 which interacts with microtubule-associated proteins 1A/1B light chain 3A (LC3) to target cargo to be enveloped by the newly forming phagophore (Mizushima et al., 2002; Gan-Or et al., 2015; Manzoni and Lewis, 2017). Moreover, cytosolic LC3-I covalently conjugates to PE, is cleaved by Atg-4 and is then converted to LC3-II before being incorporated into the autophagosome membrane. Notably, p62 and LC3 conversion are widely used as markers of autophagosome formation (Mizushima and Yoshimori, 2007; Yoshii and Mizushima, 2017). Once the phagophore completely engulfs its cargo, it forms an autophagosome, which in turn fuses with the lysosome to form the autolysosome.

The final step in autophagy is the lysosomal degradation of proteins or organelles, such as the mitochondria (designated as mitophagy), mediated by acidic lysosomal hydrolases (Mizushima et al., 2002; Manzoni and Lewis, 2017). Maintenance of acidic pH within the range of 4.5–5.0 in lysosomes is essential to activate hydrolytic enzymes and to degrade targeted cellular content (Hu et al., 2015). The low pH in lysosomes is regulated by the vATPase that pumps protons to the lysosomal lumen against their electrochemical gradient, using the energy obtained from ATP hydrolysis. A counterion flux is established to prevent the lysosome from over-acidifying, by coupling the movement of cations to the cytosol or entry of anions to dissipate the transmembrane voltage generated by the vATPase (Mindell, 2012). The TRPML1 cation channel (transient receptor potential cation channel, mucolipin subfamily, member 1) is expressed in lysosomes and late-endosomes and it releases local calcium by transporting Ca^2+^ from the lumen to the cytosol (Ghislat and Knecht, 2013; Li et al., 2017). The luminal pore structure of TRPML1 seems to be important for the Ca^2+^ and pH mediated regulation of the channel, where increased pH in lysosomes leads to decreased TRPML1 activity (Li et al., 2017). Additionally, high levels of lysosomal Ca^2+^, sustained through the maintenance of an acidic pH, are necessary for the Ca^2+^ release that precedes the fusion of lysosomes with autophagosomes or endosomes (Ghislat and Knecht, 2013; Li et al., 2017).

Considering neuronal cells are in a post-mitotic state, it is crucial that protein recycling is well maintained to ensure normal cellular and synaptic function. Furthermore, α-synuclein can be degraded via proteasome, CMA, and autophagy in neurons (Webb et al., 2003; Cuervo et al., 2004; Vogiatzi et al., 2008). Hence, impaired autophagy could cause decreased lysosomal protein degradation and lead to accumulation of aggregated α-synuclein in LBs. This proposed pathology model has been demonstrated in studies where *in vitro* treatment of neuronal cells with the inhibitor of autophagy initiation 3-MA or knocking out autophagy genes precipitated α-synuclein aggregates to accumulate in vesicle fractions, increased exocytosis of α-synuclein and transcellular transfer of α-synuclein, apoptotic cell death in the recipient cells to rise and dopaminergic axonal and dendritic degeneration to increase (Ahmed et al., 2012; Friedman et al., 2012; Lee et al., 2013). The intricate relation between LRRK2 and α-synuclein will be further addressed in the context of lysosomal function in this review.

The complexity of the ALP, combined with a broad spectrum of LRRK2 functions and the variety of PD models used, ultimately contribute to LRRK2-associated phenotypes that appear to be conflicting and seemingly difficult to integrate (Manzoni and Lewis, 2017). Our aim is to carefully review the current field of LRRK2 biology, dissecting different models and LRRK2 mutations to provide a clearer insight into the interplay of LRRK2, autophagy and lysosomal function in fibroblasts, neurons, microglia and astrocytes.

## LRRK2 in the Autophagy-Lysosome Pathway

The LRRK2 literature on autophagy is extensive and, although it is clear that LRRK2 has a role in this pathway, the exact point, and the direction in which mutations in LRRK2 affect the pathway have been referred to as uncertain or contradictory (Wallings et al., 2015; Cookson, 2016, 2017; Manzoni, 2017). Nevertheless, multiple studies have concluded that this complex protein is implicated in PD and linked to impaired autophagy and dysfunctional lysosomal activity (Manzoni et al., 2013b; Henry et al., 2015; Wallings et al., 2015, 2019; Cookson, 2017; Manzoni and Lewis, 2017; Cherubini and Wade-Martins, 2018; Schapansky et al., 2018; di Domenico et al., 2019). Therefore, it is crucial to understand the role of LRRK2 in autophagy to allow the development of new therapies.

## Phagophore Biogenesis

Once autophagy is initiated through canonical mTORC1, AMPK, or PI3K/Beclin-1 signalling pathways, the phagophore starts to encapsulate the cargo for degradation. In this early stage of autophagy, increased phosphorylation of p62 selectively binds to both ubiquitinated proteins and LC3, recruiting them to the nascent phagophore.

**FIGURE 2:**
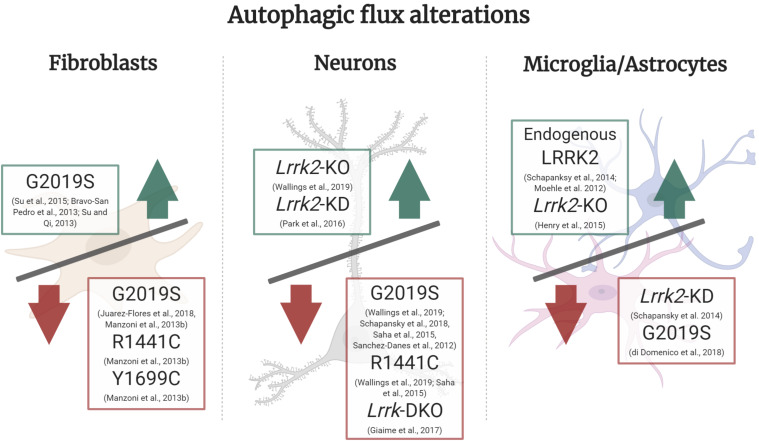
Autophagic flux alterations in different cell types and LRRK2 models. In LRRK2-G2019S fibroblasts under basal culture conditions there is an increase in autophagic flux, whereas in starvation conditions LRRK2-G2019S, R1441C, and Y1699C all show a decrease in autophagic flux. In neuronal *Lrrk2-*KO and KD models there is an increase in autophagic flux, whilst in *LRRK2-G2019S*, *R1441C* and *Lrrk-*DKO it is decreased. In microglia or astrocyte cultures, there was an increase in autophagic flux mediated by endogenous LRRK2 and in *Lrrk2-*KO models. On the other hand, *Lrrk2-*KD and *LRRK2-G2019S* overexpression cultures presented a decrease in flux. *Green upward arrow (Increase in autophagic flux)*, *Red downward arrow (decrease in autophagic flux).*

Treatment of mouse astrocyte primary cultures with LRRK2 kinase inhibitor LRRK2-in-1 has been reported to activate autophagy and increase LC3-II levels through an active Beclin-1 complex non-canonical pathway that is mTORC1 and ULK1 independent (Manzoni et al., 2016). In parallel, *LRRK2-G2019S* iPSC-derived astrocyte cultures showed increased autophagic vacuoles, decreased autophagosome-lysosome fusion and scattered, rather than perinuclear, distribution of lysosomes (di Domenico et al., 2019). Taken together, these findings imply that LRRK2 kinase activity represses autophagy in astrocytes (Figure 2).

Interestingly, prolonged inhibition of LRRK2 kinase activity in primary astrocyte cultures also showed an altered phosphorylation state of ULK1, in a mTORC1 independent manner. This uncovers a non-canonical ULK1 pathway, independent from mTORC1, that is still poorly characterised and raises the possibility that LRRK2 inhibitors for PD treatment could ultimately have the undesired effect of astrocyte autophagy dysregulation (Manzoni et al., 2018). Kalogeropulou et al. (2018) showed the cargo sequestration protein p62 has been reported to be a novel phosphorylation substrate of LRRK2 *in vitro* in human embryonic kidney 293 (HEK) cells and rat neuronal cultures. The N-terminus of LRRK2, the Ser910/935 phosphorylated residues of LRRK2 and the C-terminus of p62 are all necessary for this interaction, at which LRRK2 phosphorylates the Thr138 residue in the ZZ domain of p62. Moreover, LRRK2 mutations (N1437H, R1441C/G/H, Y1699C, and G2019S) all increase phosphorylation of p62 (Table 1). Phosphorylation of p62 was blocked when treating cells with LRRK2 kinase inhibitors MLi-2, PF745, and GNE1023. Co-expression of LRRK2-G2019S with p62 exhibited an increased neurotoxicity compared to LRRK2-G2019S with unphosphorylatable p62 (Kalogeropulou et al., 2018). However, previous work in overexpressing *LRRK2-WT* HEK models has also demonstrated that both *LRRK2-WT* and mutant *LRRK2-G2019S* indirectly reduce phosphorylation of p62, which in turn decreases its affinity to ubiquitinated cargo, thus decreasing autophagic protein degradation (Table 1). Notably, two different sites of p62 demonstrated increased phosphorylation in *LRRK2* knock-down cells, using a lentivirus-mediated shRNA *Lrrk2* knock-down in rat primary cortical neurons (Park et al., 2016). This is supported by the fact that p62 phosphorylation is associated with initiation of autophagy (Liu et al., 2016). Additionally, the relationship between LRRK2 and p62 seems to be bidirectional, where LRRK2 phosphorylates p62 (Figure 1) and p62 overexpression leads to LRRK2 degradation through the ALP. LRRK2 indirectly regulates the phosphorylation of two different residues in the SMIR domain of p62 (Ser351 and Ser403), leading to p62-mediated autophagy that degrades ubiquitinated LRRK2 (Park et al., 2016). As is apparent from the above, the interplay between LRRK2 and p62 is not fully understood as of yet and examining this will be vital to characterise its role in autophagy initiation.

**TABLE 1:** LRRK2 Models and alterations in different stages of the autophagy-lysosomal pathway.

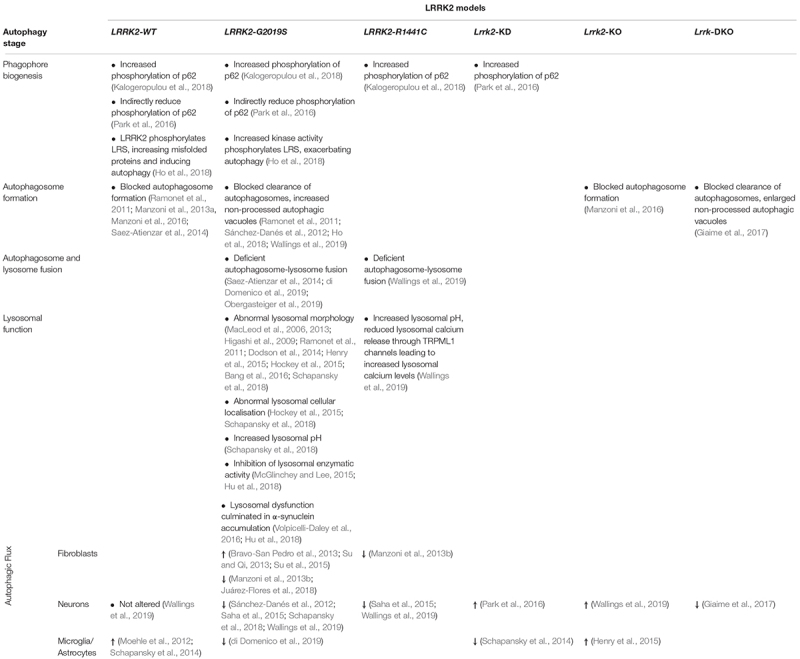

*LRS, leucyl-tRNA synthetase; ↑, *increased autophagic flux*; ↓, *reduced autophagic flux.**

Leucyl-tRNA synthetase (LRS) is a modulator of autophagy as it is responsible for the attachment of leucine to tRNA^*Leu*^, followed by activation of mTORC1 which blocks autophagy (Han et al., 2012). LRRK2 has been reported to regulate LRS by phosphorylating its conserved editing domain at residue T293, which increases the amount of misfolded proteins that accumulate, leading to ER stress and induced autophagy. Introducing the *LRRK2-G2019S* mutation increased kinase activity causing LRS to be phosphorylated, culminating in exacerbated autophagy (Figure 1; Ho et al., 2018). This unveils a new pathological pathway for LRRK2 in PD and investigation of other LRRK2 mutations should be pursued in the future to corroborate this model. Nevertheless, it seems that LRRK2 is involved in regulating autophagy for auto-degradation through interaction with p62 and could contribute to phagophore biogenesis in ER stress conditions.

## Autophagosome Formation

Following the formation of the phagophore and respective closing around the cargo, a vacuole composed of a lipidic bilayer membrane is formed, designated as the autophagosome.

LC3-I and LC3-II are present in phagophores and autophagosomes, so it is difficult to discern between these two autophagic components (Mizushima and Yoshimori, 2007; Klionsky et al., 2016; Yoshii and Mizushima, 2017). Moreover, the LC3-II/LC3-I ratio is used as an autophagy marker, however, this ratio alone does not provide sufficient data to distinguish between increased autophagosome biogenesis and reduced autophagosome clearance, therefore requiring additional immunohistochemistry techniques to properly measure this phase of autophagy (Mizushima and Yoshimori, 2007; Cookson, 2016; Klionsky et al., 2016).

A transgenic mouse model of human WT and mutant *LRRK2* overexpression, showed *LRRK2-G2019S* cultured DAn demonstrate age-dependent degeneration and also manifest an increased number of autophagic vacuoles, which reduced neurite complexity. *LRRK2-G2019S* transgenic mice also showed enlarged autophagic vacuoles *in vivo* (Ramonet et al., 2011). Yet, in neuronal cultures treated with LRRK2 kinase inhibitor LRRK2-in-1, there was an increase in LC3-II and p62 levels (Manzoni et al., 2013a). Combining LRRK2-in-1 treatment with bafilomycin A1, which blocks autophagosome-lysosome fusion by inhibiting vATPase activity, resulted in increased LC3-II levels. This suggests LRRK2 acts to block autophagosome formation (Manzoni et al., 2013a). Since then, a new generation of more specific LRRK2 kinase inhibitors have been developed, such as MLi-2, PF-06447475, and GSK2578215A (Atashrazm and Dzamko, 2016). Concomitantly, an increase in LC3B and p62 protein levels in *LRRK2-G2019S* 12–19 month-old mice brain lysate corroborates this observation *in vivo* (Ho et al., 2018). This finding was also confirmed *in vitro* using primary astrocytes cultured from a *Lrrk2* knock-out (*Lrrk2*-KO) mouse model (Manzoni et al., 2016) and by treating SH-SY5Y neurons and astrocyte cultures with the potent GSK2578215A inhibitor (Saez-Atienzar et al., 2014; Manzoni et al., 2016; Table 1). In concordance with these findings, a study in human induced pluripotent stem cell derived DAn differentiated from idiopathic or *LRRK2-G2019S*-PD patients, reported increased p62 and LC3-II levels after treatment with leupeptin and NH_4_Cl to inhibit lysosomal degradation, when compared to control DAn, indicating a blockage of autophagosome clearance (Sánchez-Danés et al., 2012). Similarly, a recent characterisation of the autophagic pathway in rat cortical primary cultures expressing human wildtype *LRRK2* (*LRRK2-hWT*) or human *LRRK2-G2019S* detected decreased levels of LC3 puncta compared to nTG after treatment with trehalose, a compound to induce lysosomal biogenesis, but no changes in LAMP1, which is a well-established lysosomal marker (Wallings et al., 2019). Hence, these data suggested that *LRRK2-hWT* and *LRRK2-G2019S* inhibit autophagosome biogenesis.

Taken together, these results demonstrate that overexpressing *LRRK2-hWT* or *LRRK2-G2019S* blocks autophagosome formation in neuronal cells (Ramonet et al., 2011; Sánchez-Danés et al., 2012; Manzoni et al., 2013a, 2016; Saez-Atienzar et al., 2014; Wallings et al., 2019), which is ameliorated by LRRK2 kinase inhibition (Manzoni et al., 2013a; Saez-Atienzar et al., 2014; Figure 2). Even though the reports mentioned in this section take advantage of different models and conditions, they consistently show increased levels of LC3-II. Thus, it is possible to speculate that both overexpression of LRRK2-WT or LRRK2-G2019S would act to block autophagosome biogenesis and clearance, giving rise to non-processed autophagic vacuoles.

## Autophagosome and Lysosome Fusion

The next step in the autophagy process is the fusion of the autophagosome with a lysosome to form the autolysosome. Recent efforts to characterise the autophagic pathway in rat cortical primary cultures expressing human *LRRK2* demonstrated an increased co-localisation of *LRRK2-R1441C* puncta to LAMP1, placing mutated LRRK2 at the lysosome. Furthermore, decreased co-localisation of LC3 and LAMP1 puncta in *LRRK2-R1441C* cultures indicates decreased autolysosome count. Hence, this suggested a deficient autophagosome-lysosome fusion in *LRRK2-R1441C* neuronal primary cultures (Figure 2; Wallings et al., 2019). Contrastingly, work using a mouse conditional transgenic model under the ROSA26 promoter in a Cre-recombinase-dependent system to selectively express *LRRK2-R1441C* in midbrain DAn did not show any abnormality in autophagic markers p62 and LC3 *in vivo* (Tsika et al., 2014). However, the authors do not reference any lysosomal markers to assess autophagic flux and this conditional transgenic model did not present any motor deficits or nigral dopaminergic neurodegeneration, contrary to other LRRK2 transgenic models (Li et al., 2009; Ramonet et al., 2011; Sloan et al., 2016).

In parallel, in a culture of iPSC-derived *LRRK2-G2019S* astrocytes, there was a decreased co-localisation of LC3 and LAMP1 in astrocytes, suggesting the autophagosome-lysosome fusion step was repressed (di Domenico et al., 2019). Together this work demonstrates that mutations in LRRK2 can affect the autophagy pathway in both neurons and astrocytes in the same way (Table 1). When exposing SH-SY5Y neuronal cultures to the LRRK2 kinase inhibitor GSK2578215A for a prolonged period, autolysosome count was also reduced (Saez-Atienzar et al., 2014). Furthermore, the impaired autophagosome-lysosome fusion reported in *LRRK2-G2019S* models might result in the presence of enlarged, dysfunctional autophagic vacuoles (Figure 1; Plowey et al., 2008). These structures could be interpreted either as abnormal large autophagosomes (Sánchez-Danés et al., 2012) or as enlarged lysosomes (MacLeod et al., 2006), which could lead to confounding conclusions on G2019S-related autophagy phenotypes. It is noteworthy to state these two reports were carried out in human iPSC-derived DAns and rat primary cortical cultures, respectively, thus this role of LRRK2 appears to be conserved across the two species and cell types.

Work by Manzoni et al. (2016) concluded that a LRRK2-dependent increase in autophagosomes was not caused by a decline in autophagosome-lysosome fusion but by an increase in autophagosome biogenesis. Although, a caveat of that study was that measurement of autophagosomes (LC3-II/LC3-I) and lysosomal function was conducted in H4 neuroglioma cell cultures. Caution should be taken given that autophagy has been reported to be upregulated in H4 neuroglioma cancer cells to overcome stress induced apoptosis (Zhou et al., 2016), unlike in PD neuronal models where autophagy is deficient. On the other hand, a study by Obergasteiger et al. (2019) using SH-SY5Y cells overexpressing *LRRK2-WT* or *LRRK2-G2019S* shows that *LRRK2-G2019S* neurons present with increased LC3-II protein levels and LC3B puncta, indicating an autophagosome accumulation. Measurement of autolysosome production using a double-tagged GFP-LC3-mCherry construct in this model showed autolysosomes were reduced in *LRRK2-G2019S* when compared to *LRRK2-WT*, suggesting a defective autophagosome-lysosome fusion (Figure 1). Proteolytic activity of lysosomes assessed with DQ-Red-BSA assay demonstrated LRRK2-G2019S induced a defect in lysosomal activity, culminating in an accumulation of endogenous α-synuclein inclusions. Defects in autophagosome-lysosomal fusion, proteolytic impairment and α-synuclein accumulation phenotypes were rescued after treatment with the PF-475 LRRK2 kinase inhibitor, highlighting the importance of LRRK2 in autophagy-mediated α-synuclein degradation (Obergasteiger et al., 2019).

Hence, there is robust evidence that demonstrates the two most common LRRK2 mutations, G2019S and R1441C, lead to an obstruction of the fusion between autophagosomes and lysosomes and result in the presence of large dysfunctional autophagic vacuoles.

## Lysosomal Function

After the autolysosome is formed, the maintenance and regulation of lysosomal function, including correct lysosomal acidity, is crucial for protein degradation and recycling. It is important to maintain the lysosomal pH at around 4.5–5.0 as hydrolytic enzymes in the lysosome are only active at a narrow acidic pH interval (Hu et al., 2015). Investigation of *LRRK2-R1441C* cortical primary cultures demonstrated lysosomal pH was significantly less acidic in these neurons and consequently autophagosome-lysosome fusion and lysosomal protein degradation were decreased (Wallings et al., 2019). Furthermore, *LRRK2-R1441C* neuronal cultures also demonstrated significantly increased intracellular calcium stores driven specifically by increased lysosomal calcium levels. In addition, *LRRK2-R1441C* cultures were shown to have significantly reduced lysosomal calcium release through TRPML1 channels in the lysosomal membrane (Table 1). Calcium release from lysosomes is a key step for the autophagosome-lysosomal fusion and also for the late endosomal-lysosomal fusion (Ghislat and Knecht, 2013). This work also revealed a novel interaction between LRRK2 and subunit a1 of the proton pump vATPase, for which the encoding gene *ATP6V0A1* is also a GWAS hit for increased risk in PD (Chang et al., 2017). This interaction was severely decreased in the *LRRK2-R1441C* neurons (Wallings et al., 2019).

Several authors have reported enlarged lysosomes in different *LRRK2-G2019S* models, including SH-SY5Y neurons, transgenic mice models, primary cortical neurons, primary astrocytes, and human *post-mortem* tissue (MacLeod et al., 2006, 2013; Higashi et al., 2009; Ramonet et al., 2011; Dodson et al., 2014; Henry et al., 2015; Hockey et al., 2015; Bang et al., 2016) as well as in *LRRK2-R1441C* and *LRRK2-Y1699C* mouse primary astrocytes (Henry et al., 2015). Enlarged lysosomes also appeared to be perinuclear and aggregated in *LRRK2-G2019S* derived fibroblasts and were normalised after treatment with LRRK2 kinase inhibitors. This abnormal lysosomal morphology was also rescued by blocking TPC2, an endo-lysosomal ion channel implicated in Ca^2+^ signalling from acidic organelles (Hockey et al., 2015).

Primary cortical neurons from a *LRRK2-G2019S* knock-in mouse model also described similar lysosomal phenotypes as *LRRK2-R1441C* rat primary cortical neurons described above, where lysosomal pH was aberrantly increased exceeding physiological values (Schapansky et al., 2018; Wallings et al., 2019). In addition, perinuclear and distal lysosomal count was increased while lysosomal size was decreased in *LRRK2-G2019S* neurons, reflecting altered lysosomal function. Reported lysosomal dysfunction was associated with accumulation of endogenous, detergent-insoluble α-synuclein and increased neuronal α-synuclein release into the media (Schapansky et al., 2018; Table 1). In parallel, a model of *LRRK2* overexpression in HEK cells has demonstrated that LRRK2-G2019S inhibits the activity of lysosomal enzymes Cathepsin B and L that play a vital part in lysosomal α-synuclein degradation (McGlinchey and Lee, 2015). This debilitated lysosomal function lead to inhibition of the lysosomal degradation of α-synuclein, promoting its aggregation. This mechanism could underlie LRRK2 and α-synuclein pathology, characteristic of PD. Given the *LRRK2-G2019S* induced inhibitory effect on Cathepsins B and L was not dependent on kinase activity (Hu et al., 2018), it would be interesting to verify this finding in other LRRK2 mutations, in addition to confirming it in neuronal cell models. A transcriptomic analysis of autophagy related genes in SH-SY5Y cells overexpressing *LRRK2-WT* or *LRRK2-G2019S* also found altered mRNA levels of *CTSB*, which encodes for Cathepsin B (Obergasteiger et al., 2019). Overexpression of *LRRK2-G2019S* in cultured neurons and DAn in the rat SNpc exhibited an accumulation of α-synuclein inclusions after exposure to sonicated α-synuclein fibrils, when compared to nTG and to *LRRK2-WT*. These α-synuclein inclusions decreased after treatment with two separate LRRK2 kinase inhibitors, rescuing the accumulation of α-synuclein in the *LRRK2-G2019S* neurons and implicating LRRK2 kinase activity in the observed phenotype. Additionally, Volpicelli-Daley et al. (2016) have demonstrated that *LRRK2-WT* overexpression did not induce α-synuclein inclusions. Collectively, these studies suggest a putative role of LRRK2 in α-synuclein accumulation.

Nonetheless, the interaction between LRRK2 and α-synuclein remains unresolved, with some reports being inconsistent. For instance, while one study concluded that inhibiting endogenous LRRK2 kinase activity by incorporating MLi-2 into the diet of an α-synuclein PFF PD mouse model did not protect neurons from α-synuclein pathology or motor deficits (Henderson et al., 2019), another study showed that administering the HG-10-102-01 LRRK2 kinase inhibitor intraperitoneally to transgenic mice overexpressing human WT α-synuclein significantly reduced trans-axonal α-synuclein aggregates and phosphorylated α-synuclein in different brain regions of transgenic mice (Bae et al., 2018). However, the results presented by Henderson et al. (2019) and Volpicelli-Daley et al. (2016) taken together could suggest that it is mutated LRRK2 that increases the progression of pathological α-synuclein inclusions by increasing a pool of α-synuclein that is more susceptible to forming inclusions. These observations along with studies demonstrating α-synuclein is processed by lysosomes in cell models overexpressing LRRK2 (Hu et al., 2018; Obergasteiger et al., 2019), underline the prospects of targetting lysosomal function as novel drug-developing avenues worth pursuing for LRRK2-related PD.

To summarise, evidence has been accumulating for the key role of LRRK2 and lysosomal function in PD. The studies discussed above indicate that the LRRK2 mutations G2019S and R1441C present altered lysosomal pH and consequently affect lysosomal activity and protein degradation (MacLeod et al., 2006, 2013; Higashi et al., 2009; Ramonet et al., 2011; Dodson et al., 2014; Henry et al., 2015; Hockey et al., 2015; Bang et al., 2016; Hu et al., 2018; Schapansky et al., 2018; Wallings et al., 2019). Indeed, lysosomal markers LAMP1, Cathepsin D, and HSP73 are decreased in PD nigral tissue, as well as increased α-synuclein aggregates, when compared to age-matched controls (Chu et al., 2009). A recent report described how repurposing clioquinol, an anti-parasitic drug, could be used to revert autophagic impairment and lysosomal dysfunction phenotypes in a neuronal *LRRK2-R1441C* model, demonstrating the ability to modulate such phenotypes (Wallings et al., 2019).

Therefore, it will be important to further investigate the role of mutated LRRK2 in lysosomal maturation, maintenance of lysosomal pH, and lysosomal calcium homeostasis in the future.

## Regulation of the Autophagic Flux

In this section we will focus on several LRRK2 models that strongly suggest both excessive and diminished LRRK2 activity can lead to impaired autophagic flux (Schapansky et al., 2014). In turn, either an exacerbated or insufficient autophagic flux could lead to neurodegeneration which is observed in PD pathology (Chu, 2006). Specifically, disturbances in autophagy impose downstream effects on neurons, such as accumulation of non-degraded α-synuclein and its respective release into extracellular media (Henry et al., 2015; Obergasteiger et al., 2018; Schapansky et al., 2018; di Domenico et al., 2019), reduced neurite outgrowth (MacLeod et al., 2006) and impaired mitophagy (Su and Qi, 2013; Su et al., 2015). These PD phenotypes caused by dysfunctional ALP indicate autophagy and lysosomal activity could, in part, be underlying the molecular basis for this neurodegenerative disorder. Abnormal LRRK2 function can lead to impaired autophagy and lysosomal function due to a disruption in the cellular autophagic flux, yet different cell types present with distinct cellular bioenergetic needs and protein turnover rates. Accordingly, in efforts to create a clear picture of the role of LRRK2 in autophagic flux, we individually review the autophagic flux in fibroblasts, neurons and microglia/astrocyte LRRK2 models (Figure 2).

Currently, one of the main challenges in the LRRK2 autophagy field is the measurement of autophagic flux. Since autophagic flux is a dynamic process occurring within cells, it is crucial to utilise a combination of methods that would enable an interpretation of the several different stages of autophagy, from phagophore biogenesis to lysosomal degradation of cargo. Techniques such as measurement of autophagic markers by western blot have limitations. For instance, the autophagic marker LC3 is expressed throughout different stages of autophagy, making it difficult to interpret autophagic alterations by analysing LC3 changes alone. Thus, autophagic marker measurement would normally represent a single time point in the autophagic flux. By combining these approaches with immunofluorescence, co-localisation and pH sensitive dyes, it is possible to obtain a more accurate picture of the autophagic flux.

### Fibroblasts

Fibroblasts can be obtained from patients in a safe, non-invasive and inexpensive manner and have been widely used in reprogramming techniques to obtain iPSCs, which in turn can be differentiated into iPSC-derived neurons (Bahmad et al., 2017). Despite being a non-neuronal model, fibroblasts are useful cellular models to investigate and predict PD pathology.

In fibroblasts isolated from patients with *LRRK2-R1441C* and *LRRK2-Y1699C* mutations (located in the ROC-COR domains) undergoing starvation, there was a decrease in WIPI2 and p62 puncta and a decrease in LC3-II/LC3-I ratio, suggesting a decreased autophagic flux (Figure 2). In the case of *LRRK2-G2019S* isolated fibroblasts under starvation conditions, cells showed a decrease in LC3-II/LC3-I ratio while WIPI2 and p62 levels were not significantly altered compared to wildtype fibroblasts. It is important to add that immunoblot for LAMP1 did not show differences in any of the mutations studied, indicating a disruption of autophagy upstream of the lysosomes. However, this finding was not confirmed with immunocytochemistry techniques (Manzoni et al., 2013b). When comparing fibroblasts from skin biopsies of *LRRK2-G2019S* individuals not manifesting PD symptoms, *LRRK2-G2019S* PD patients and healthy controls, non-manifesting *G2019S* demonstrated upregulated autophagy, and preserved mitochondrial function while fibroblasts from *LRRK2-G2019S* PD patients presented with elevated p62 levels, reduced LC3-II ratios and mitochondrial dysfunction. Thus implicating exhaustion of mitochondrial bioenergetic and autophagic reserve in the development of PD (Juárez-Flores et al., 2018). Collectively, these studies indicate decreased autophagic flux in *LRRK2-G2019S* fibroblasts (Figure 2; Manzoni et al., 2013b; Juárez-Flores et al., 2018).

On the other hand, in a separate study PD *LRRK2-G2019S* fibroblasts had decreased p62, but showed an increase in Beclin-1, LC3, LAMP1, and Cathepsin B, culminating in an increase of autophagic flux and lysosomal activity, in basal conditions (Figure 2 and Table 1; Bravo-San Pedro et al., 2013). The authors use LAMP2 as a lysosomal marker and levels were reported to be increased (Bravo-San Pedro et al., 2013). However, LAMP2A has been implicated in CMA (Bandyopadhyay et al., 2008), and since LAMP1 was not measured it could be hypothesised that the increase in autophagic flux can represent an increase in CMA specifically, rather than macroautophagy.

A similar model reported excessive autophagic flux in the context of increased mitophagy in primary fibroblasts from *LRRK2-G2019S* PD patients, as a consequence of mitochondrial depolarisation and dysfunction (Su and Qi, 2013; Su et al., 2015). It is important to note the studies had different autophagy cellular contexts (basal vs. starvation) and fibroblasts were cultured in slightly different conditions. Mutated LRRK2 autophagic phenotypes have been described as contradictory (Cookson, 2016; Manzoni, 2017; Manzoni and Lewis, 2017). However, since LRRK2 physiological function in fibroblasts is not completely understood, perhaps it is not entirely unexpected that *LRRK2-G2019S* would display different results under distinct conditions, once again highlighting the importance of comparing between similar cell types and culture conditions, for instance basal *vs.* starvation.

It is equally important to adopt a consistent method for autophagic flux analysis. If the same markers and conditions were to be applied across the field in a more conventional and consistent manner, it would become easier to compare and interpret the literature. Other cell types, for instance HEK and PC12 (rat pheochromocytoma cells from the adrenal medulla) overexpressing human LRRK2, have shown an increased autophagic flux in the presence of *LRRK2-WT* and *LRRK2-G2019S*, through Ca^2+^-dependent activation of a CaMKK/adenosine monophosphate (AMP)-activated protein kinase pathway (Gómez-Suaga et al., 2012). Nonetheless, these cellular models do not entirely recapitulate neurodegeneration and would need further confirmation.

### Neurons

Given the clear loss of SNpc DAn in PD, research has focused on understanding the neuronal role of LRRK2 through numerous models. Analysis of autophagic markers in *Lrrk2-*KO primary cortical neurons demonstrated no significant changes in p62 and LAMP1 levels, yet showed increased LC3-II conversion and importantly, increased lysosomal protein degradation, when compared to WT using the pulse chase assay (Wallings et al., 2019). Contrary to previous *Lrrk2-KO* literature conveying there were autophagic changes in the kidney but not in the brain (Tong et al., 2012), this indicates an increase in autophagic flux in *Lrrk2-*KO neurons (Figure 2; Wallings et al., 2019). However, a novel double *Lrrk* knock-out (DKO) mouse model in which both *Lrrk1* and *Lrrk2* are deleted, meaning LRRK1 does not compensate for the lack of LRRK2, reported a decrease in autophagic flux in neurons (Figure 2). This was evidenced by increased p62, decreased LC3-I and increased LC3-II levels in different sub-regions of the *Lrrk*-DKO mouse brain, including SNpc and striatum. Quantitative EM analysis also demonstrated age-dependent accumulation of autophagosomes and autolysosomes in the SNpc of *Lrrk*-DKO mouse, and consequent autophagy impairment. Interestingly, autophagic dysfunction was observed at 10 months and preceded accumulation of α-synuclein and dopaminergic neurodegeneration (seen at 15 months), indicating disrupted autophagy can lead to PD pathology (Giaime et al., 2017). When comparing *Lrrk2*-KO mouse model phenotypes with *LRRK2* mutant models some similarities emerge, with the autophagosome processing stages of autophagy being particularly affected (Table 1). While *Lrrk2*-KO shows blocked autophagosome biogenesis, *Lrrk*-DKO, *LRRK2-G2019S*, and *LRRK2-R1441C* show blocked clearance of autophagosomes into autolysosomes (Ramonet et al., 2011; Sánchez-Danés et al., 2012; Manzoni et al., 2016; Giaime et al., 2017; Ho et al., 2018; di Domenico et al., 2019; Obergasteiger et al., 2019; Wallings et al., 2019). Given LRRK2 is a complex protein involved in several different functional processes in the cell, considering mutations in LRRK2 in a strict binary loss or gain of its normal function might not reflect the full scope of the LRRK2 mutant effects in its downstream pathways.

As discussed above, *Lrrk2* knock-down (*Lrrk2-*KD) in rat primary cortical neurons exhibit increased p62 phosphorylation (Park et al., 2016), which in turn promotes phagophore biogenesis and is associated with autophagy initiation. Nevertheless, this study did not measure other important autophagic markers such as LC3 and LAMP1.

Early evidence in cultured HEK cells where *LRRK2* was knocked-down with siRNA showed increased turnover of lipidated LC3, measured by the LC3-I and LC3-II ratio, which reflected an increased autophagic activity (Alegre-Abarrategui et al., 2009). However, the lack of evidence on the effects of neuronal LRRK2*-*KD in autophagy means further validation is necessary to conclude whether it induces an increase in autophagic flux. Comprehensive characterisation of autophagy and lysosomal function in rat primary cortical neurons expressing human *LRRK2-G2019S* and *LRRK2-R1441C* has revealed this pathway is compromised whereas those from *Lrrk2*-KO rats demonstrate an upregulation of autophagic flux. In the case of *LRRK2-hWT* and *LRRK2-G2019S* neurons, there was an inhibited autophagosome production, while in *LRRK2-R1441C* expressing neurons there was a decreased autophagosome-lysosome fusion and lysosomal dysfunction (Wallings et al., 2019). In agreement with these findings, a non-neuronal model overexpressing *LRRK2-R1441C* in HEK cultures also described that cells displayed accumulation of large autophagic vacuoles, increased p62, and decreased protein degradation, which translated into an impaired autophagic balance (Alegre-Abarrategui et al., 2009). This shows similar phenotypic alterations in autophagy in two separate studies and cell types. Measuring lysosomal protein degradation using the pulse-chase assay, arguably the most accurate method for measuring true autophagic flux, uncovered an overall decreased lysosomal degradation across the LRRK2 genotypes, with the *LRRK2-R1441C* resulting in the most significant alteration. In parallel, there was also an increase in LC3 puncta *in vivo*, in *LRRK2-R1441C* DAn of the SNpc of 22-month-old animals, when compared to nTG. Consequently, neuronal autophagic flux was decreased in *G2019S* and *R1441C* mutations (Wallings et al., 2019). Equally, mouse primary neuronal cortical cultures overexpressing *LRRK2-G2019S* showed decreased LC3-I and LAMP1 levels and increased basal levels of LC3-II. The decreased LC3-I and LAMP1 levels were also confirmed *in vivo*, in 20-month-old *LRRK2-G2019S* knock-in mouse cortical tissue. *LRRK2-G2019S* neurons had increased number yet smaller lysosomes that were mis-localised and had less acidic pH. This points to poor lysosomal activity and decreased autophagosome turnover in *LRRK2-G2019S* neurons and consequently, a significantly decreased autophagic flux. ALP disruption increased α-synuclein accumulation and release from neurons, which were rescued with the LRRK2 kinase inhibitor GSK2578215A (Schapansky et al., 2018).

Research into DAn derived from iPSCs of familial PD patients with the *LRRK2-G2019S* mutation and of idiopathic PD patients, cultured during a prolonged period (up to 75 days) induces stress conditions that mimicked *in vivo* ageing in patients. These iPSC-derived DAns showed decreased LC3 flux and co-localisation of LC3/LAMP1, accumulated autophagic vesicles and decreased lysosomal function when compared to healthy controls, thus indicating a reduced autophagic flux (Figure 2). *LRRK2-G2019S* neurons also showed a decreased number and length of neurites (Sánchez-Danés et al., 2012). Similarly, analysis of autophagic markers in the basal ganglia of *LRRK2-G2019S* patient *post-mortem* tissue showed a decrease in p62 and LAMP1 in comparison to matched idiopathic PD patients, assessed both by immunohistochemistry and immunoblotting (Mamais et al., 2018). Furthermore, overexpression of human *LRRK2-WT* in *C. elegans* DAn improved autophagy throughout their life span whereas *LRRK2-G2019S* and *LRRK2-R1441C* expression inhibited autophagy (Figure 2). *LRRK2-G2019S* expression accelerated age-related loss of autophagic function and co-expression of either mutant or WT LRRK2 with α-synuclein further accentuated inhibition of autophagy and DA neuronal death (Saha et al., 2015). In agreement, various other models have indicated *LRRK2-G2019S* leads to impaired autophagy and lysosomal function, such as follicle cells in Drosophila Melanogaster (Dodson et al., 2012, 2014), human neuroepithelial stem cells (Walter et al., 2019) and HEK cells (Hu et al., 2018). Induced protein quality control-associated autophagy was also impaired in both SH-SY5Y neurons and transgenic mice overexpressing LRRK2-G2019S (Bang et al., 2016).

Attempting to draw conclusions from LRRK2 and its role in ALP has resulted in conflicting reports. In the present review, after analysing both *LRRK2-G2019S* patient derived iPSC models and models overexpressing *LRRK2-G2019S* there is consensus that the G2019S mutation leads to a decrease in autophagic flux (Sánchez-Danés et al., 2012; Manzoni et al., 2013b; Saha et al., 2015; Juárez-Flores et al., 2018; Schapansky et al., 2018; di Domenico et al., 2019; Wallings et al., 2019; Table 1). This change is likely attributed to an increase in kinase activity as evidenced by rescue of autophagic flux when using LRRK2 kinase inhibitors (Manzoni et al., 2013a; Saez-Atienzar et al., 2014; Wallings et al., 2019).

Since LRRK2 function is not yet fully understood, it is important to investigate the role of wildtype LRRK2 in autophagy under physiological conditions in neuronal cell systems. To this effect, KD and KO studies of LRRK2 revealed an increased autophagic flux (Park et al., 2016; Wallings et al., 2019). Interestingly, contrary to *Lrrk2-*KD in neuronal cells, *Lrrk2-*KD in microglia suggested deficits in the induction of autophagy, although only one autophagy marker was analysed along with protein clearance, as discussed below in this review (Schapansky et al., 2014). This highlights once more the degree of LRRK2 phenotypic cellular heterogeneity. Furthermore, a *Lrrk2-*KO mouse model detected phenotypic alterations in the kidneys and lungs but not in the brain (Tong et al., 2012, 2010). This could be due to a compensating mechanism in the brain, as suggested by the decrease in the flux of autophagy present in the *Lrrk-*DKO mouse model (Giaime et al., 2017). Moreover, a decreased autophagic flux was also reported in LRRK2 knock-in or transgenic models (Saha et al., 2015; Schapansky et al., 2018; Wallings et al., 2019; Table 1). Considering LRRK2 regulates several functions in the cell, it is important to investigate LRRK2-mediated alterations in autophagic flux in the context of other cellular organelles and even other cell types, as any processes depending on autophagy will likely be perturbed. These processes could include lysosomal function as well as synaptic vesicle trafficking and recycling, mitophagy and the endo-lysosomal pathway (Pan et al., 2017; Wang, 2017; Connor-Robson et al., 2019).

It seems LRRK2 acts as a brake in the ALP in neuronal cells, which is supported by an increase in autophagic flux when LRRK2 is knocked-out (Park et al., 2016; Wallings et al., 2019). However, when LRRK1 and LRRK2 are simultaneously knocked-out, or when LRRK2 is mutated, there is a reduced autophagic flux (Sánchez-Danés et al., 2012; Saha et al., 2015; Giaime et al., 2017; Schapansky et al., 2018; Wallings et al., 2019), which points to the conclusion that LRRK2 function is also required for normal autophagic function to some extent. This argument suggests associating LRRK2 with loss-of-function in autophagy might be contradictory and unclear. Other authors have also discussed the issue of gain vs. loss of function in LRRK2-associated PD and concluded that it is not a straightforward concept in LRRK2 literature (Gan-Or et al., 2015; Cookson, 2017).

Thus, LRRK2 function and potential regulation of autophagy remain unclear. Future studies will be necessary to validate this hypothesis and to elucidate the role of LRRK2 in neuronal autophagy.

### Microglia and Astrocytes

Investigating neuronal models exclusively does not recapitulate the intricacies of the human brain environment. Neurons are surrounded and supported by microglia, astrocytes and oligodendrocytes (collectively referred to as glial cells) and form a complex network that likely play a role in any phenotypic outcome.

In non-neuronal models such as microglia BV2 cultures and through staining of glial cells in mouse brain tissue, endogenous LRRK2 expression increases upon microglia activation (Figure 2; Moehle et al., 2012; Schapansky et al., 2014; Lee et al., 2017). This leads to LRRK2 phosphorylation, translocation and recruitment to autophagosomal membranes, which drives an increase in autophagy. Upon stimulation of microglia with Toll-like-Receptor 4, increasing autophagy and phagocytosis could be an anti-inflammatory defense mechanism in the context of neuroinflammation (Deretic, 2011; Schapansky et al., 2014). On the other hand, KD of LRRK2 in microglia shows deficits in the induction of autophagy, a direct effect of a decreased LC3-II conversion ability in these cells, and in autophagic protein clearance after rapamycin treatment (Figure 2; Schapansky et al., 2014). However, this study did not measure multiple autophagy markers, such as p62 or LAMP1 to confirm altered autophagic flux. Still, this supports a model of PD pathology where LRRK2 regulates autophagy in microglia differently to its role in neurons.

Research into neuron-astrocyte co-culturing systems has also contributed to our understanding of LRRK2 PD pathology. By generating co-cultures of iPSC-derived astrocytes and ventral midbrain DAn from either familial *LRRK2-G2019S* patients or healthy individuals, it was revealed that *LRRK2-G2019S*-derived astrocytes accumulate α-synuclein and co-cultured control vmDAns display shortened neurites and neurodegeneration (di Domenico et al., 2019). Additionally, *LRRK2-G2019S* vmDAns co-cultured with control-derived astrocytes showed less severe neurite shortening, a more complex neurite arborisation and decreased α-synuclein accumulation in neurons, when compared to co-cultures with *LRRK2-G2019S* astrocytes. This effect was independent of direct neuron-astrocyte contact. However, the authors do not show autophagy characterisation in co-cultured neurons or astrocytes (di Domenico et al., 2019).

Turnover of α-synuclein is processed by both CMA and autophagy (Xilouri et al., 2013), which were both impaired in *LRRK2-G2019S* astrocytes. *LRRK2-G2019S* derived astrocytes revealed an increased number of autophagosomes (LC3-positive puncta) that were localised in both distal and perinuclear regions, opposed to a preferable mainly perinuclear distribution. However, a decreased LC3/LAMP1 co-localisation, higher p62 and reduced LC3-II levels revealed an autophagosome-lysosome fusion blockage, resulting in deteriorated autophagic flux in *LRRK2-G2019S* astrocytes (Figure 2; di Domenico et al., 2019). In agreement to the findings described in microglia models, astrocyte models unveil a key role for glial cells in non-cell autonomous PD pathogenesis. In this scenario, a non-cell autonomous LRRK2-mediated increase in lysosomal secretion may increase α-synuclein release and aggregation, augmenting PD pathology. This is supported by observing the release of lysosomal contents into the cytosol when exposing cells to lysosomal overload stress, where Rab7L1, LRRK2, and phosphorylated Rab8/10 are sequentially accumulated onto the stressed lysosomes (Eguchi et al., 2018). However, primary astrocyte cultures from *Lrrk2-*KO mouse showed no difference in lysosomal size compared to nTG, whilst lysosomal count almost doubled. Thus, it could indicate autophagic flux is increased in *Lrrk2-*KO, concomitant with lysosomal dysfunction and in agreement with studies of *Lrrk2-*KO neuronal cultures (Figure 2). The authors report variable effects of LRRK2 manipulations in autophagy when examining LC3 and p62 levels but data was not shown in the report (Henry et al., 2015). As discussed above, measurement of autophagic markers would be fundamental to determine whether LRRK2*-*KO induces autophagic flux alterations in astrocytes. Complementary to this, characterisation of an array of autophagic-lysosomal markers in WT and *Lrrk2*-KO mouse bone marrow-derived macrophages, after infection with *Mycobacterium tuberculosis*, revealed an increase in autophagic flux wherein LAMP1-positive phagosome as well as Cathepsin L-positive phagosome count is increased, but there are no differences in p62 and LC3B (Härtlova et al., 2018).

As evidenced in the sections above, focusing only on measuring markers of autophagosome formation might not be sufficient to infer on the dynamics of autophagic flux, since altered levels of autophagosomes and autolysosomes do not necessarily implicate impaired lysosomal activity or protein degradation (Giménez-Xavier et al., 2008; Cookson, 2017). Hence, it would be extremely valuable to combine the measurement of an array of proteins to assess autophagic flow in future research, including autophagosome, autolysosome and lysosomal markers as well as treatment with autophagy modulators such as 3-MA, bafilomycin and chloroquine (Klionsky et al., 2016; Yoshii and Mizushima, 2017). Investigating the late phases of autophagy is also a crucial part of monitoring autophagy, including lysosomal function and protein degradation assays to measure degradation of autophagic substrates, such as pH-sensitive tagged proteins, Lysotracker dye to analyse lysosomal morphology, LysoSensor dye to measure lysosomal pH and pulse-chase assay to measure protein degradation (Klionsky et al., 2016; Yoshii and Mizushima, 2017).

Nevertheless, the LRRK2-G2019S mutation seems to have a similar impact in activated microglia when compared to fibroblasts and neurons, resulting in a decreased autophagic flux (Table 1). By partially reducing LRRK2 expression and function using siRNA/shRNA, there is an increased induction of autophagy, except in activated microglia where autophagy decreases. In activated microglia cultures, endogenous LRRK2 expression increases and there is an increase in autophagic flux. Therefore, it is possible that LRRK2 positively regulates the autophagy machinery in the context of neuro-inflammation. However, the mechanism underlying this regulation in glial populations is still unknown and more research should be focused to resolve LRRK2 and its contribution to autophagy in PD.

## Conclusion and Perspectives

In the present review we have interrogated the current LRRK2 literature to elucidate how this protein is involved in regulating the ALP and we would emphasise three main concluding remarks.

First, as discussed above, different PD models have informed on the different mechanisms whereby LRRK2 mutations impact on its functional activity and can lead to disease (Wallings et al., 2015; Cookson, 2016, 2017; Manzoni and Lewis, 2017; Cherubini and Wade-Martins, 2018; Connor-Robson et al., 2019). Therefore, the use of different PD models and systems has firstly revealed that LRRK2 phenotypes display cellular heterogeneity, which is an important consideration for future studies of LRRK2 function (Schapansky et al., 2015). Indeed, LRRK2 mutations manifest different pathogenesis depending on the cell type (Zeng et al., 2018). Therefore, some caution should be exercised when selecting cultural conditions (basal vs. starvation) and comparing LRRK2 models to study autophagy in PD, as well as considering human versus non-human LRRK2 expression, as it is paramount to confirm any phenotypes in biologically relevant contexts of disease. Hence, in this review we have also individually assessed autophagic flux in fibroblasts, neurons and microglia/astrocytes. Concurrently, it will be crucial to combine neuronal and non-cell autonomous methods, for instance by utilising 3D midbrain cultures or co-cultures of neuronal and neuroimmune cells, in the efforts to completely understand LRRK2 pathology in PD.

Second, it is essential to point out that accurate measurement of autophagic flux is crucial when comparing LRRK2 phenotypic effects in the ALP. Analysing LC3-I and LC3-II could provide insights into the rates of autophagosome formation, however, LC3 is present in phagophores, autophagosomes and autolysosomes and measurement would often only represent a single time point, rather than the flow of autophagy. Another widely used marker of autophagy is LAMP1, which is expressed in lysosomes and autolysosomes but also in late endosomes of the endocytic pathway. This could introduce confounding factors in studies relying solely on one specific marker.

And finally, upon thorough dissection of LRRK2 phenotypes relating to autophagic flux, it is evident that LRRK2 mutations, specifically the G2019S and R1441C, act in a different manner and at different stages of the autophagy pathway (Table 1). This observation may not be surprising given these mutations are situated in different enzymatic domains of LRRK2, the kinase domain and GTPase domain, respectively (Figure 2). The most common LRRK2 mutation, G2019S, is consistently associated with an increase in kinase activity and a decrease in autophagic flux (Sánchez-Danés et al., 2012; Manzoni et al., 2013b; Saha et al., 2015; Juárez-Flores et al., 2018; Schapansky et al., 2018; di Domenico et al., 2019; Wallings et al., 2019) whereas the second most common mutation, R1441C, exhibits decreased autophagic flux, with lysosomal activity being particularly impaired (Saha et al., 2015; Wallings et al., 2019). Conceivably, impaired lysosomal function could lead to deficient protein clearance and could fit the model of Lewy body pathology wherein there is an accumulation of aggregated α-synuclein (Orenstein et al., 2013; Schapansky et al., 2015, 2018; Hu et al., 2018). Indeed, impaired autophagy has been recently proposed to be an aggravator of PD (Johnson et al., 2019). Consequently, different LRRK2 mutations located in different domains will have distinct cellular effects downstream and alongside of autophagic impairment, such as dysregulated mitophagy, endocytosis and vesicular trafficking (Manzoni et al., 2013b; Chen and Wu, 2018; Connor-Robson et al., 2019).

Ultimately, research into the relation of LRRK2 and lysosomal activity could have an impact on new drug screening approaches to potentially find novel compounds to treat LRRK2 related PD.

## Author Contributions

MM planned and wrote the manuscript, and composed the figures. NC-R planned, edited, and supervised the writing of the manuscript. RW-M supervised and reviewed the manuscript.

## Conflict of Interest

The authors declare that the research was conducted in the absence of any commercial or financial relationships that could be construed as a potential conflict of interest.

## Abbreviations

3-MA: 3-methyladenine
ALP: autophagy-lysosomal pathway
AMP: adenosine monophosphate
AMPK: adenosine monophosphate-activated protein kinase
ANK: ankyrin repeat
ARM: armadillo repeat
ATG: autophagy-related gene
CMA: chaperone-mediated autophagy
COR: C-terminal of Roc
DA: dopamine
DAn: dopaminergic neurons
DKO: double knock-out
EM: electron microscopy
ER: endoplasmic reticulum
GCase: glucocerebrosidase
GWAS: genome wide association studies
HEK: human embryonic kidney 293
hWT: human wild type
iPSCs: induced pluripotent stem cells
KD: knock-down
KO: knock-out
LAMP1: lysosome-associated membrane protein 1
LAMP2: lysosome-associated membrane protein 2
LAMP2A: lysosome-associated membrane protein 2 isoform A
LBs: Lewy bodies
LC3-I: microtubule-associated protein 1 light chain 3 beta isoform 1
LC3-II: microtubule associated protein 1 light chain 3 beta isoform 2
LRR: leucine-rich repeat
LRRK1: leucine-rich repeat kinase 1
LRRK2: leucine-rich repeat kinase 2
LRS: Leucyl-tRNA synthetase
mTORC1: mammalian target of rapamycin complex 1
nTG: non-transgenic cultures
PD: Parkinson’s disease
PE: phosphatidylethanolamine
PI(3)P: phosphoinositol-3-phosphate
PI3K: phosphoinositol 3 kinase
PINK1: PTEN-induced putative kinase 1
ROC: Ras of complex
SNpc: substantia nigra pars compacta
TRPML1: transient receptor potential cation channel mucolipin subfamily member 1
ULK1: uncoordinated-51-like kinase 1
vATPase a1: vacuolar type H^+^-ATPase a1 subunit
vATPase: vacuolar type H^+^-ATPase
vmDAns: ventral midbrain dopaminergic neurons
VPS34: vacuolar protein sorting 34
WIPI: WD repeat domain phosphoinositide-interacting
WT: wild type.
